# New insights into honey bee (*Apis mellifera*) pheromone communication. Is the queen mandibular pheromone alone in colony regulation?

**DOI:** 10.1186/1742-9994-7-18

**Published:** 2010-06-18

**Authors:** Alban Maisonnasse, Cédric Alaux, Dominique Beslay, Didier Crauser, Christian Gines, Erika Plettner, Yves Le Conte

**Affiliations:** 1 INRA, UMR 406, Abeilles et Environnement, Laboratoire Biologie et Protection de l'Abeille, Site Agroparc, 84914, Avignon, France; 2 INRA, UMR 408 Sécurité et Qualité des Produits d'Origine Végétale, Site Agroparc, 84914, Avignon, France; 3 Department of Chemistry, Simon Fraser University, 8888 University Drive, Burnaby, B.C. V5A 1S6, Canada

## Abstract

**Background:**

In social insects, the queen is essential to the functioning and homeostasis of the colony. This influence has been demonstrated to be mediated through pheromone communication. However, the only social insect for which any queen pheromone has been identified is the honey bee (*Apis mellifera*) with its well-known queen mandibular pheromone (QMP). Although pleiotropic effects on colony regulation are accredited to the QMP, this pheromone does not trigger the full behavioral and physiological response observed in the presence of the queen, suggesting the presence of additional compounds. We tested the hypothesis of a pheromone redundancy in honey bee queens by comparing the influence of queens with and without mandibular glands on worker behavior and physiology.

**Results:**

Demandibulated queens had no detectable (E)-9-oxodec-2-enoic acid (9-ODA), the major compound in QMP, yet they controlled worker behavior (cell construction and queen retinue) and physiology (ovary inhibition) as efficiently as intact queens.

**Conclusions:**

We demonstrated that the queen uses other pheromones as powerful as QMP to control the colony. It follows that queens appear to have multiple active compounds with similar functions in the colony (pheromone redundancy). Our findings support two hypotheses in the biology of social insects: (1) that multiple semiochemicals with synonymous meaning exist in the honey bee, (2) that this extensive semiochemical vocabulary exists because it confers an evolutionary advantage to the colony.

## Background

A remarkable trait of social insect colonies is the assemblage of individuals into a coherent social unit. Members of the society exhibit an organization mainly controlled by a complex pheromonal language [1]. Behavioral evidence for division of reproduction and labor in the colony indicates the importance of pheromones in both queen-worker and worker-worker interactions, including mediating the regulation of task allocation [2]. In the case of honey bees, coordination of the different tasks is partly mediated by chemical signals [2]. In social insects pheromones provide the colony with a rich syntax that is important for the spread of information and the integration of social behavior.

In honey bees, even though some workers can lay eggs, the queen produces most of the eggs and is the progenitor of several thousand bees in a colony. In addition she provides central information that regulates colony homeostasis, growth and reproduction [3]. "Queen substance", (E)-9-oxodec-2-enoic acid (9-ODA) is a queen pheromone produced in the mandibular glands and that was the first identified honey bee pheromone with functional roles in the colony [4]. Later, in 1988 Slessor et al. [5] discovered four other compounds from the mandibular glands that act synergistically with 9-ODA: both enantiomers of 9-hydroxydec-2-enoic acid (9-HDA), methyl p-hydroxybenzoate (HOB) and 4-hydroxy-3-methoxyphenylethanol (HVA). These five chemicals constitute QMP, which strongly attracts young workers and stimulates queen tending (feeding, licking and antennating the queen). When these young workers subsequently interact with other bees, the QMP is dispersed throughout the colony by antennation, cuticular contacts and trophallaxis between the workers [6]. In 2003, Keeling et al. discovered four other compounds that synergize with QMP for retinue behavior, in particular in bees that do not respond strongly to QMP with retinue behavior [7].

The other main function of QMP is the inhibition of worker ovary activation [8]. Reproductive control is essential to colony stability and functionality since reproductive workers do not work as efficiently as normal worker bees [9]. QMP also controls comb construction by stimulating quantitative and qualitative worker-sized cell construction [10]. It inhibits the construction of drone and queen cells [11] until colony growth results in a less efficient QMP distribution [12]. New QMP functions are still being discovered; for example, besides mediating worker behavioral maturation [13], QMP also increases resistance to starvation [14] and affects olfactory learning and memory [15].

QMP is thus integrated into colony life as a powerful and central systemic regulator. However, QMP does not control the full gamut of behavioral and physiological responses that result from the presence of a queen. For example, Velthuis and Van Es [16,17], found that queens from which mandibular glands were removed still retained their regulatory functions. Their experiments demonstrated that the mandibular glands are not essential for inhibition of queen cell construction, retinue behavior and inhibition of worker ovary activation. However, it is not clear from their studies whether the demandibulated queens triggered the full worker response that is triggered by intact queens. The effect of demandibulated queens on a colony was not directly compared to colonies headed by intact queens or to queenless colonies. The exception was worker ovary activation, which showed almost the same effect with intact as with demandibulated queens [17]. Consequently, others sources of queen pheromone have been proposed including tergal, tarsal and Dufour's glands [2,18]. A series of studies demonstrated that Dufour extracts attracted workers [19] and tergal glands affected both ovary activation and retinue behavior [20,21]. However a queen has ca. 0.5 μg (out of ca. 150-200 μg total) of 9-ODA on her cuticle surface [22] and previous studies did not check for the presence of QMP residues in Dufour and tergal gland extracts or in queens without mandibular glands [19-21]. Without a control for QMP residue one could hypothesize that the effects of the different experiments on worker control could be due to those pheromone residues. Thus, the relative contribution of other queen chemicals besides QMP is not well understood and the following question remains unanswered: In addition to the well-known pheromone pleiotropy of the QMP, do queens also use different pheromones that converge on the same function (pheromone redundancy)?

To answer this question, we investigated the importance of additional queen pheromones by surgically removing the mandibular glands from virgin queens and checking for QMP residue on the queen bodies. We then asked whether demandibulated queens were as effective as normal queens in regulating ovary activation, comb construction and retinue behavior. A regulatory control as effective as a normal queen would demonstrate that additional queen chemicals might be as important as QMP in regulating colony functionality and thus support the hypothesis of pheromone redundancy.

## Methods

### Honey bee queen rearing

Experiments were performed in Avignon (France) in 2005, 2007 and 2009 with local colonies derived from populations of a mixture of European subspecies of *Apis mellifera *(*A. m. ligustica *and *A. m. mellifera*). Queen rearing was performed according to standard beekeeping methods [23]. One day before hatching, queen cells were removed from their hive and placed individually in cages in an incubator (34°C, 60% RH) with 10 day-old workers. They were fed *ad libitum *with water, candy (30% honey from the source colonies, 70% powdered sugar) and pollen. One-day-old bees were obtained from honey combs containing last-stage pupae removed from 3 source colonies. In each replicate, queens originated from the same colony to reduce genetic variation and thus potential pheromone variation [24,25].

### Dissection of mandibular glands

Mandibular gland excision was performed using a method modified from Gary [26] when queens were one or two-days old, since mandibular glands do not secrete chemicals outside the body until 3 days after emergence [27]. Experimental queens were narcotized lightly with CO_2 _(~15 seconds) and placed under a binocular magnifying glass (×8), kept on the back between the thumb and forefinger in order to clear the head. Mandibles were carefully removed with scissors and forceps by cutting the articulation of the mandibles. An opening appeared on both sides of the mouth. Then, the mandibular glands were carefully extirpated from the queen heads with extra fine forceps. After surgery, the demandibulated queens (MG-) were returned to their own cage. One day later, the mandible incisions had healed. Control queens (MG+) were sham operated by the same procedure, except mandibular gland extirpation.

### Pheromone analysis

The presence of queen mandibular pheromone components (9-ODA-HOB-HVA-9-HDA) in MG- (n = 17) and MG+ (n = 19) queens was analyzed at the end of the 2009 experiment. Queens were individually stored at -20°C for later chemical analysis of the QMP components. Head, thorax and abdomen were dissected and extracted separately in 200 μl of methanol and 100 μl of decanoic acid (250 ng/μl; internal standard). Preparations were cooled on ice, body parts were crushed with a glass rod for 2 minutes and centrifuged (2500 × g for 20 min. at 4°C). The supernatant was collected, the total volume of supernatant recorded and a sample (20 μl) was concentrated under a nitrogen stream and then derivatized with 5 μl of bistrimethylsilyltrifluoroacetamide (BSTFA). The solution was agitated and left at room temperature for 40 min. The derivatized sample was then diluted in 100 μl of isohexane and 1 μl of this solution was injected into a fast gas chromatograph (Shimadzu 2014, Japan) equipped with a split-splitless inlet, a flame ionization detector, and a capillary column (equity-5; 15 m × 0.10 mm, 0.10 μm film thickness). The samples were injected in split mode. Hydrogen was used as the carrier gas with column flow of 0.52ml min^-1^. The oven temperature was set at 100°C, then 100°C to 200°C at 40°C min^-1 ^and 200°C to 250°C at 10°C min^-1 ^and held at 250°C for 2 min. Standard solutions of each QMP compounds derivatized with BSTFA were used to calibrate the response of the instrument with respect to the internal standard. Identification and quantification of HOB, 9-ODA, HVA, 9-HDA were based on retention times of synthetic compounds (Sigma-Aldrich, France and PheroTech, Canada) and on the internal standard method. The confirmation of QMP compounds was done by a mass spectrometer (Shimadzu CP2010, Japan). The mass spectrometer was operated in the electron impact mode at 70 eV with continuous scans (every 0.2 sec) from a mass to charge ratio (m/z) of 70 to 400. Data were collected with GC-MS Solution software (Shimadzu, Japan). Compounds were identified by comparison with standards. The variation in QMP amount between the MG- and MG+ queens was statistically determined, compound by compound, using Mann-Whitney U tests (STATVIEW 5.0, SAS Institute, Cary, NC).

### Experimental set up

The effect of MG- and MG+ queens on both ovary activation and comb construction was tested in cage experiments. Plastic cages (11 × 8.5 × 5.8 cm) [28] were composed of 150 one day-old bees originating from 3 colonies and fed *ad libitum *with water, pollen (to promote ovary activation), and candy. They were kept in an incubator (33°C and 60% RH) during 15 days and were then collected for ovary activation analysis. Ovary activation generally reaches a peak at 14-15 days in cage [29]. A piece of wax (5 × 1 cm) was stuck on the top of the cage as primer for comb cell construction. Three different groups were tested: cages with a normal queen (MG+: positive control), queenless cages (QL: negative control), and cages with a demandibulated queen (MG-). Since queens emit highly volatile chemicals [30], each group was separated in different incubators with the same environment.

### Ovary activation

Twenty bees reared in QL or MG+ or MG- conditions were randomly collected from each cage for ovary activation analysis. They were dissected under a binocular microscope, and the level of ovary activation was classified into 5 stages according to Pernal and Currie [31] as follows: stage 0: no follicle development, ovaries are slender and non-differentiated, referred to undeveloped ovaries, stage 1: slight enlargement, beginnings of differentiation; stage 2: presence of distinct cells leading to swellings and constrictions, stage 3: egg volume exceeding that of the nutritive follicle, stage 4: presence of fully formed eggs, ovaries are characterized by having mature oocytes and referred to fully formed ovaries. The dissector was *blind *to the treatment identity of bees. One repetition (2009) was performed with 55 cages (MG-: n = 17, MG+: n = 19 and QL: n = 19). The MG-, MG+ and QL effects on worker ovary activation stage was determined using a Kruskal-Wallis ANOVA test followed by Mann-Whitney U post-hoc tests.

### Comb construction

At day 15, the comb construction from each cage was collected and the number of cells counted. The mean diameter of 20 cells/cage/treatment was determined and divided into two categories according to their size, worker-sized cells' diameters being from 5 to 5.4 mm and drone-sized cells from 6.2 to 6.4 mm [3]. In addition, the number of royal draft cells, which are conical and elongated, was counted in the different groups. Three repetitions (2005, 2007, and 2009) were performed giving a total of 125 cages (MG-: n = 53, MG+: n = 36 and QL: n = 36). Queen treatments effect on cell number and size were analyzed using a two-way ANOVA (repetitions and treatments) followed by Fisher post-hoc tests. The number of cells was transformed: y' = ln(y + 1) to attain variance homogeneity in the 3 groups.

### Retinue behavior

The effects of queens MG-, MG+ on retinue behavior were analyzed in two one-frame standardized observation hives containing 3,000 one day-old bees. For each repetition, one day-old bees were collected from the same hives. Each hive was established as similar as possible with one frame containing equivalent proportion of honey, pollen, brood and eggs. Hives were placed in an indoor apiary (25°C) and connected to the outside to allow normal foraging activity. The queens were not allowed to mate and introduced into the hive 20 days after hatching. Two days after queen introduction in the observation hives, a series of 5 pictures were taken twice. The number of workers surrounding the queen was determined and used to estimate retinue behavior. Then the queen was replaced randomly by a new queen MG- or MG+. One repetition (2009) was performed giving a total of 15 replicates for both MG- and MG+ queens. The number of bees performing the retinue behavior was compared by using a Mann-Whitney U test.

## Results

### Pheromone analysis

**Figure 1:**
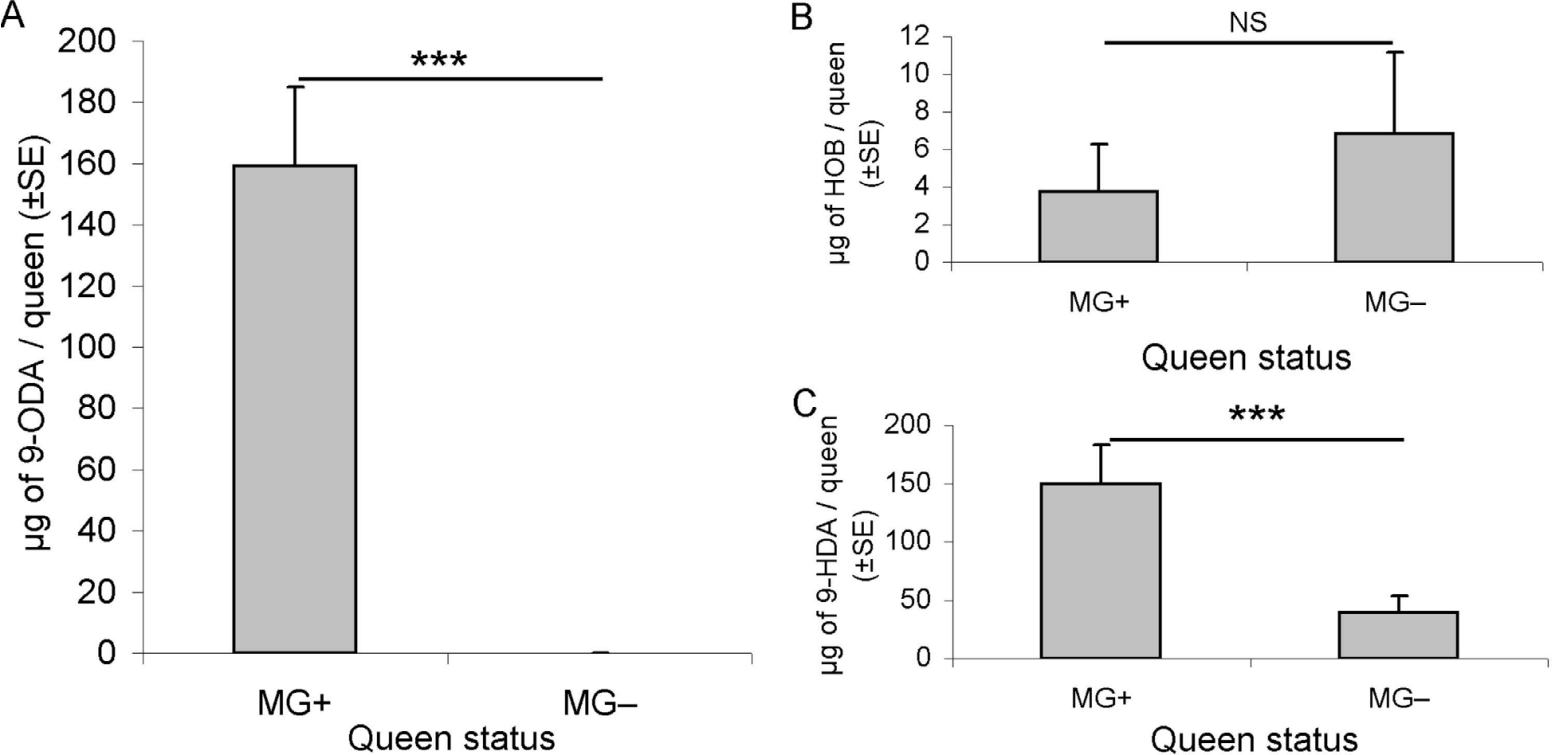
**Levels of QMP components in control and demandibulated queens**. (a) 9-ODA (b) HOB (c) 9-HDA. *** denotes significant differences (P < 0.001) and NS: Non significant difference between treatments. MG+: control queen, MG-: demandibulated queen, QL: queenless.

Normal amounts of 9-ODA (159 ± 26 μg), HOB (3.7 ± 2.5 μg) and 9-HDA (150 ± 34 μg) were found in queen MG+ [10]. As found by Ledoux et al (2001) [10], HVA was not detected in virgin queens. Interestingly, quantities of 9-HDA (39 ± 14 μg) and HOB (7 ± 4 μg) were detected in queen MG-, 9-ODA was not detectable (minimum GC detection equal at 0.47 ng of 9-ODA/μL of isohexane) (Fig. 1). As a result, 9-ODA was only found in queen MG+ (Z = -5.05, P < 0.0001); 9-HDA was higher in quantity in queen MG+ compared to queen MG- (Z = -3.5, P < 0.0005) but there was no significant difference in the amount of HOB between the two queen types (Z = -1.13, P = 0.25).

### Ovary activation

**Figure 2:**
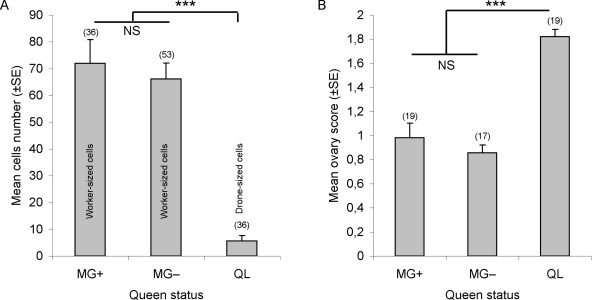
**Effect of queen treatment on (a) cell construction and (b) worker ovary activation**. Data show mean number of cells ± SE and ovary score ± SE. Number of cages are indicated in parenthesis. *** denotes significant differences (P < 0.001) and NS: Non significant difference between treatments. MG+: control queen, MG-: demandibulated queen, QL: queenless.

We found a significant treatment effect on worker ovary activation (N = 1100, H = 102.1, df = 2, P < 0.0001, fig. 2). Bees reared with queen MG+ or MG- had a significantly lower ovary activation compared to bees from QL cages (MG- vs. QL: Z = -9.34, P < 0.0001; MG+ vs. QL: Z = -9.04, P < 0.0001). However, despite differences in pheromone composition, the effect of queens MG+ and MG- on worker ovary activation did not differ significantly (Z = -0.737, P = 0.5). The percentage of workers in MG-, MG+ and QL cages, respectively, with no ovary activation (range 0-1) was 82%, 81% and 52%, and workers with ovary activation (range of 3-4) was 3%, 4% and 28%.

### Comb construction

We found significant treatment and repetition effects on comb construction, but no interaction effect between the two factors (treatment: F_2,124 _= 121.8, P < 0.0001, repetition : F_2,124 _= 12.6, P < 0.0001, treatment × repetition : F_4,249 _= 1.18, P = 0.32). The comb size (number of cells) significantly increased in the queen presence (MG+, MG-) compared to QL cages (MG+ vs. QL: P < 0.0001, MG- vs. QL: P < 0.0001), however no differences were detected between the two types of queen (MG+ vs. MG-: P = 0.68, Fig. 2). The queen treatment also had an effect on the cell size (F_2,124 _= 130.8, P < 0.0001). This effect did not change between repetitions (F_2,124 _= 1.92, P = 0.15). Workers reared with MG+ and MG- queens built worker-sized cells that did not differ significantly in their diameters (5.13 ± 0.07 and 5.20 ± 0.06 mm, respectively; P = 0.94) but QL workers built drone-sized cells that were larger (6.15 ± 0.08 mm; MG+ vs. QL: P < 0.0001, MG- vs. QL: P < 0.0001).

No royal cell construction was observed in our experimental set-up with either queens MG+ or MG-. However, QL workers constructed one to three royal draft cells per cage (1.3 ± 0.2).

### Retinue behavior

The mean number of workers performing retinue behavior around queens MG- and MG+ reached 10.3 ± 0.5 and 10.7 ± 0.2, respectively and was not significantly different (Z = -0.38, P = 0.7).

## Discussion

Previous investigations found that pheromones from mandibular glands have a pronounced effect on colony life [18]. Due to QMP importance, it was expected, that queens from whom mandibular glands were removed would be less effective in regulating worker responses. Our results do not support this hypothesis but show that demandibulated queens retain their full regulatory functions (Table 1), highlighting some redundancy in queen control. Our results are in accordance with the studies of Velthuis and Van Es [16,17], suggesting that QMP is not responsible by itself for the queen's pheromonal regulation of colony function (worker ovary activation, queen cell construction and retinue behaviour). This phenomenon can now be extended to the regulation of general comb construction (cell number and type) (this paper). In addition, by checking for the first time the effect of mandibular gland removal on the composition of 9-ODA, 9-HDA and HOB, we showed that demandibulated virgin queens were as effective as normal virgin queens in regulating colony function.

**Table 1:** Comparative effect of queenless (QL), control queen (MG+), and extirpated queen (MG-) on worker behavior and physiology.

	MG+	MG-	QL
Worker ovary inhibition	+	+	-
Retinue behavior	+	+	Ø
Cells construction	+	+	-
Cells type	♀	♀	♂
Queen cells inhibition	+	+	-

(♀ worker cells construction, ♂ drone cells construction, Ø not available, + positive, - negative)

Consistent with previous studies [25,32], sham-operated queens (MG+) had normal levels of QMP. Moreover in this study, queens from whom mandibular glands had been removed (MG-) had a similar levels of HOB, lower levels of 9-HDA and no detectable 9-ODA. This confirms that 9-ODA is uniquely produced and stored in the queen mandibular glands [22] and suggests the existence of another source of production of HOB and 9-HDA as found by Whiffler and Hepburn [33] in *A. m*. *capensis *and *A. m. scutellata *queens.

Queens produce a blend of 9 compounds (Queen Retinue Pheromone, QRP) that, in concert, elicit almost the full queen retinue behavior from honey bee workers. Pure 9-ODA can elicit weak queen retinue behavior, whereas the other compounds act synergistically with 9-ODA and do not elicit a retinue response by themselves [5,7]. This pheromone blend is composed of QMP, coniferyl alcohol produced in the mandibular glands and 3 other compounds, methyl oleate, hexadecan-1-ol and linolenic acid, produced in the body of the queen [7]. Contrary to our expectation, and despite no 9-ODA detectable, MG- queens had a similar number of workers performing retinue behavior (around 10) compared to the sham-operated control queens (between 8 to 12 workers [3,34]). Therefore, as methyl oleate, hexadecan 1-ol and the linolenic acid are not produced in the mandibular gland [7] and 9-HDA and HOB are found in MG- queens, those compounds might play a role together or with other, as yet non-identified, components in eliciting retinue behavior.

Our results confirm that the two types of virgin queen, MG- and MG+, partially inhibit ovary activation in workers. Thus, other queen-produced substances have the potential to substitute for 9-ODA. Recently, a volatile compound, E-*β*-ocimene, was found to be produced by mated queens [30] and larval brood [35], and this compound has been found to inhibit ovary activation in workers [35]. But E-*β*-ocimene was not found in 3 day-old virgin queens [30]. In our experiment virgin queens were 5 to 20 days old, thus complementary experiments are needed to know if virgin queens older than 3 days could produce this compound or if mating is required to increase the production of this compound, as is the case for HVA [25].

Furthermore, virgin and mated queens produce esters [36], such as ethyl palmitate (EP), which have the potential to suppress ovary activation in workers [37]. EP works efficiently at 5400 ng per bee and the queen produces only 330 ng of EP, thus EP emission by the queen could act in addition to larval EP production or other queen chemicals but is unlikely to act alone in mediating ovary inhibition. Tergal gland extracts can also partially regulate ovary activation in workers [21], but the presence of 9-ODA on the queen's cuticle [6] might be involved. In addition, the effect of 9-HDA and HOB together or separately was not tested on worker ovaries, however their inhibitory action in the QMP blend has been documented. It is possible that E-*β*-ocimene, ethyl palmitate, compounds from tergal glands, HOB and 9-HDA act in synergy to provide a full worker response similar to normal queens.

Interestingly, workers with a MG- queen produced worker-sized cells, and built a large number of cells, as in the MG+ queen condition, in contrast to the QL condition in which workers constructed a small number of cells that were drone-sized. Thus, our results indicate that comb construction is also regulated by queen chemicals other than QMP [10]. In the absence of the queen, *A. m. capensis *workers, who reproduce via thelytokous parthenogenesis and *A. m. scutellata*, who reproduce via arrhenotokous parthenogenesis build only worker or drone cells, respectively, but queenless hybrid colonies produce both cell types or only worker cells [38]. This would support the idea that comb construction can be regulated by chemicals other than QMP that are also produced by the workers. However, since *A. m. capensis *workers develop QMP-profiles with a high amount of 9-ODA [39], the construction of worker cells in those queenless colonies could also be due to the QMP.

This study used virgin queens, however mating in honey bee queens causes dramatic changes in queen behavior and physiology [40]. For example, the queen pheromone blend is modulated by the reproductive status of the queens. Virgin and newly mated queens produce the same QMP signal [41] while a different QMP blend is produced by the mature mated queen [25]. Therefore, whether demandibulated mated queens keep their regulatory functions, like virgin MG- queens, remains to be tested.

The evidence for multiple, active queen compounds with similar effects raises the question of why such redundancy? An answer to this question may be found in the theoretical analysis of communication in social insects. Two opposing theories can potentially explain the evolution of pheromone communication between the queen and workers. On one hand it is believed that the queen pheromone acts as a reliable and honest signal, to which workers respond by restraining themselves from reproducing in order to increase their inclusive fitness, but on the other hand, queen pheromones could be used to control and manipulate worker reproduction [42,43]. This dishonest control over reproduction by the queen would be evolutionarily unstable, because workers would be selected to overcome her inhibitory effect. As a consequence, workers would be selected for a reduced sensitivity to specific queen chemicals, to which the queen would develop an alternative pheromone source. In that case, queen pheromone would evolve towards a multi-component blend, as opposed to a relatively simple, honest single-component signal [42,43]. The redundancy of multiple, active queen compounds might be the result of competition between queens and workers over reproduction [44-46]. Differences in sensitivity to QMP between colonies [47] and evidence of workers being able to lay eggs that can survive, despite the inhibitory presence of a queen [48,49], are both found in nature. This shows that workers have the capacity to bypass queen pheromonal control of reproduction. Since, *A. m. capensis *parasitic workers, who reproduce despite the presence of a queen, develop a QMP-profile [39,50,51] to compete pheromonally with the host queen or workers, it would be interesting to determine whether they have also developed multiple, redundant queen chemicals other than QMP-like.

A second and alternative explanation to the pheromone redundancy hypothesis would be that the presence of multiple queen pheromones might fine-tune the regulation of colony homeostasis. The different queen chemicals may have redundant functions, but their efficiency may differ and depend on the context, their transmission [18] and the variability in their production. In summary, each chemical may not be effective by itself, but altogether, they enable the queen to develop a complex and precise chemical "syntax" during the colony life-cycle. In addition, worker behavior and physiology is regulated by multiple hormone signaling pathways (e.g. juvenile hormone, vitellogenin, insulin) [52-54], so it is possible that the different but redundant queen chemicals each act on different targets of the worker hormonal system.

## Conclusion

Queen-worker communication is essential to colony homeostasis. For the past 20 years, 9-ODA, and consequently QMP, were described as the main regulatory system of worker behavior and physiology. Now, our results demonstrate that other queen chemicals as powerful as 9-ODA and QMP are involved in worker regulation. Now the next challenge is to find the secondary queen pheromonal system and test for its effects on the hormonal system. In honey bees, pheromone signaling systems have pleiotropic effects as regulators of colony functionality. The signal redundancy originating from the same individual now adds another level of complexity to the already intricate language of the colony.

## Competing interests

The authors declare that they have no competing interests.

## Authors' contributions

AM, EP, YLC designed the experiments. AM, DB, DC performed the experiments with the honey bees. AM, CG, DB carried out the chemical analyzes and analyzed the chemical data. AM, CA analysed and interpreted the data. AM, CA, YLC, EP wrote the manuscript. All authors read and approved the final manuscript.
